# Physically Crosslinked Conductive Organic Gel with Excellent Elasticity and Environmental Stability

**DOI:** 10.3390/gels12080707

**Published:** 2026-08-08

**Authors:** Haiquan Zhang, Zhinan Zhao, Shishen Lan, Qiadong Yao, Minglei Lv, Ning Wang

**Affiliations:** State Key Laboratory of Marine Resource Utilization in South China Sea, School of Marine Sciences, Hainan University, Haikou 570228, China

**Keywords:** organogels, stretchability, electrical conductivity, wearable electronic skin

## Abstract

Liquid water in hydrogels exhibits an adsorption-desorption dynamic equilibrium with the surrounding environment, which leads to the instability of mechanical properties. To address this limitation, we propose an innovative design of conductive composite organogels by incorporating compatible linear lauryl alcohol (LA) and multi-walled carbon nanotubes (CNTs) into a poly(butyl methacrylate) (PBMA) network. Carbon chains of LAform physical crosslinks with PBMA side chains, effectively replacing inherent polymer chain entanglements. This structural innovation facilitates rapid chain rotation and sliding during stretching, so that the gel has a super stretching property of up to 2460%. At elevated temperatures, weakened interactions between LA–PBMA and PBMA–PBMA chains reduce physical confinement of CNTs within the PBMA network. Simultaneously applying a directional electric field, CNTs undergo rotation and translation to reconstruct an optimized conductive pathway, granting the composite distinctive temperature-sensitive electrical conductivity. Critically, all components in the PBMA/LA/CNTs (PLCs) exhibit low volatility and hydrophobicity. These characteristics enable the organogel to retain excellent flexibility and stable electrical performance after prolonged immersion in deionized water, exposure to vacuum, and even under extreme conditions at 120 °C. Such comprehensive stability suggests promising applications in deep-sea exploration and aerospace engineering.

## 1. Introduction

In recent years, stretchable polymer hydrogel materials with excellent ion conductivity have attracted extensive attention and have been successfully applied to temperature, pressure and strain sensors [1,2]. The inherent stretchability of these materials primarily originates from non-covalent intermolecular interactions, including hydrogen bonding, ionic bonds [3,4], van der Waals forces [5,6], π–π stacking [7,8], and electrostatic interactions [9,10]. However, soft materials relying on a single type of non-covalent interaction often suffer from insufficient mechanical strength, typically demonstrating fracture stresses below 100 kPa. To synergistically achieve high stretchability and exceptional mechanical strength, researchers have engineered polymeric hydrogel systems with sophisticated microarchitectures, such as hydrophobically associated hydrogels [11,12], interpenetrating/semi-interpenetrating double-network hydrogels [13,14], nanocomposite hydrogels [15,16], and topological hydrogels [17,18]. Due to the copolymerization of acrylamide (AAM) and acryloylpluronic 127 micelles, the fracture strain of the block copolymer hydrogel was optimized to 1500 kPa [19]. Zhang et al. [20] introduced water-soluble lithium terephthalate and self-dispersing stannous terephthalate nanoparticles into a hydrogen-bonded PAM network, resulting in the PAM/PTALi/PTASn composite exhibiting a fracture strain of 6640% and a fracture stress of 670 kPa. Joshi et al. [21] recently constructed alginate/carbon nanotube composite conductive hydrogel through 4D printing technology, showing a good application prospect in the field of flexible strain sensing and neural conduit.

Water molecules intrinsically bound within hydrogel polymer networks inevitably undergo exchange with the ambient environment until adsorption-desorption equilibrium is attained. Crucially, the mechanical properties of hydrogels are directly dictated by their water content. A reduction in moisture content significantly diminishes stretchability while precipitously increasing toughness [22,23]. For example, the PAM-based hydrogel changed from a superelastic soft material with a fracture strain of 3500% to a white rigid material with little water content when it was placed at 25 °C with a relative humidity of 65% for 24 h [24]. On the other hand, sub-zero temperatures readily induce crystallization of the liquid aqueous phase, causing hydrogel embrittlement. Although conventional strategies employ water-soluble additives (e.g., ethylene glycol [25], glycerol [26]) to enhance freeze-thaw resistance, such approaches merely decelerate the dynamic water exchange process between the hydrogel and its surroundings rather than fundamentally eliminating it. Consequently, the long-term stability and sustainability of hydrogel mechanical performance under realistic operational conditions present substantial challenges.

While PBMA-based organic gels have been investigated for years, the vast majority of existing studies only employ PBMA as a copolymerization component or auxiliary additive. Representative reported cases include the fabrication of multi-network organic hydrogels via copolymerization with PLMA [27], the preparation of electrode materials by compositing nanogels with conductive polymers [28], and the development of hydrophobic low-temperature gels for drug delivery [29]. To date, there has been no published report on the construction of conductive organic gels that use PBMA homopolymer as the primary network through physical crosslinking strategies.

Additionally, organic gels based on paraffin oil, ionic liquids or deep eutectic solvents have been extensively explored as stretchable conductive materials, yet they are inherently restricted by their respective drawbacks. Paraffin oil-based systems are prone to phase separation under mechanical tension or elevated temperatures due to weak intermolecular interactions [30]. Ionic liquid gels, which rely on ionic conduction mechanisms, show performance highly vulnerable to moisture and temperature fluctuations [31]. Deep eutectic solvent gels, whose structural integrity is excessively maintained by hydrogen bonds, tend to dissociate under high humidity or extreme temperature conditions [32].

**Figure 1:**
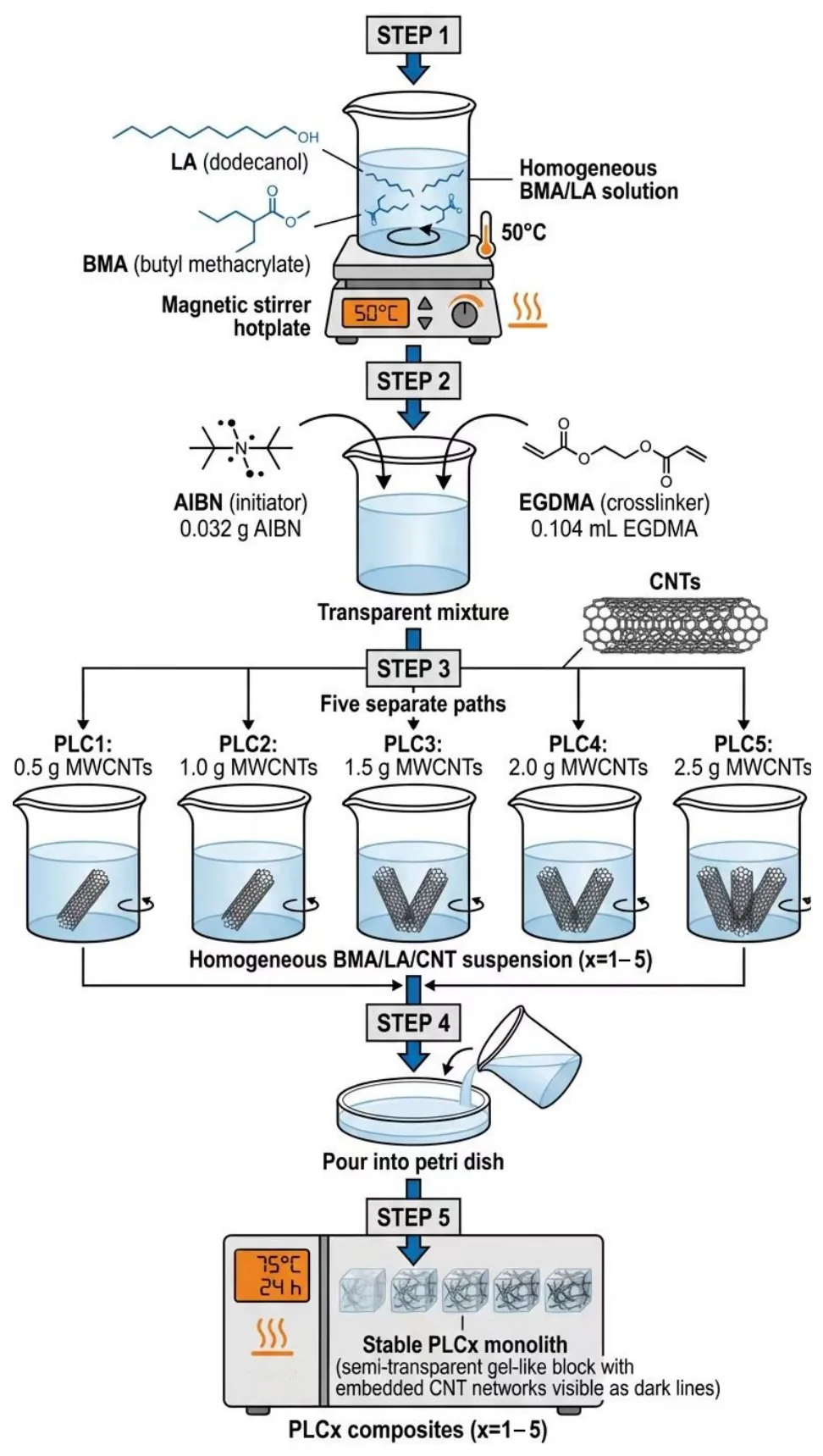
Schematic diagram of preparation of highly elastic oil-based gel.

Inspired by biomimetic principles and composite hydrogel design paradigms, this study proposes a novel composite oleogel strategy. We constructed an elastic, mechanically robust, and conductive oleogel composite by integrating hydrophobic LA and CNTs into a PBMA network governed by van der Waals interactions (Figure 1). Benefiting from the favorable compatibility between LA and the butyl methacrylate (BMA) monomer, physical crosslinking forms between the LA backbone and PBMA side chains. This interaction effectively mitigates the inherent entanglement effect within the PBMA skeleton, imparting a plasticizing effect. Consequently, unlike intrinsically rigid PBMA polymer, the PBMA/LA oleogel manifests a fracture strength of approximately 120 kPa coupled with an exceptional fracture strain of ~2460%. Simultaneously, the dispersed carbon nanotubes within the PBMA/LA/CNTs composite gel exhibit a synergistic response to both temperature and electrical fields, rendering the composite a promising candidate for thermally sensitive electronic components. More critically, the PLC composite gel demonstrated reliable flexibility and stable electrical performance under demanding conditions, including immersion in deionized water, exposure to vacuum, or sustained operation at 120 °C. This work draws inspiration from biological elastic soft tissues, such as mammalian dermis and muscle fibers, as the biomimetic prototypes. These natural systems construct dynamic physical crosslinking networks via reversible noncovalent interactions (e.g., van der Waals forces and hydrophobic interactions), which enable reversible slippage and reconfiguration of molecular chains under external forces. Meanwhile, embedded neural sensing units are introduced to synergistically perceive multiple external stimuli including deformation, temperature, and electric potential, while the surface hydrophobic lipid barrier is leveraged to suppress water exchange, ensuring long-term stability of both mechanical and sensing performances in complex environments.

## 2. Results and Discussion

### 2.1. Design of the PL and PLC Oleogels

Based on the principles of bioimetics and the design strategies for composite hydrogels, this study employed linear aliphatic alcohols (ROH) in the polymerization of butyl methacrylate to successfully synthesize a series of superelastic materials. The preparation was rather simple process of mixing butyl methacrylate, lauryl alcohol, and multi-walled carbon nanotubes (mass ratio of 5:2:1:20) with a zobisisobutyronitrile (AIBN) chain initiator and ethylene glycol dimethacrylate (EGDMA) crosslinking agent, followed by thermal-induced polymerization of BMA at 75 °C for 24 h. The polymer oleogel’s structure was guided by two key design principles: molecular chain entanglement modulation for elasticity enhancement and the dynamic construction of a conductive network. The incorporation of liquid ROH effectively disrupted the inherent strong interchain side-chain entanglement within the PBMA, significantly lowering the energy barrier for chain movement. This granted the resulting oleogel’s exceptional stretchability. Concurrently, applying an external electric field to the CNTs medium induced their directional alignment along the field. This field-induced orientation enabled the composite’s electrical conductivity to increase reversibly with higher field strengths.

A notable feature is the competitive dynamic equilibrium between ROH–PBMA and PBMA–PBMA physical crosslinks. This balance is highly sensitive to temperature, shifting distinctly towards stronger PBMA–PBMA intermolecular interactions as temperature decreases. Upon cooling below a specific critical temperature, the electric field loses its ability to dynamically orient the CNTs confined within the PBMA network, resulting in the complete disappearance of the conductivity response. Consequently, the composite’s conductivity exhibits changes spanning an order of magnitude under the dual stimulus of electric field and temperature, establishing it as an outstanding temperature-sensitive electrical sensing material.

In-depth studies reveal that the alkyl chain length (R) of alkanol molecules critically regulates their interaction strength with the PBMA matrix. When decanol (C10) is employed, the polymerization of BMA is completely inhibited, preventing the formation of a viable polymer network. Immersing PBMA polymers in nonanol (C9) induces pronounced swelling and eventual dissolution of the matrix. Excessively long alkyl chains, however, markedly weaken alkanol–PBMA interactions. For instance, the tetradecanol (C14)/PBMA composite transitions from a rigid material to a transparent superelastic state when heated above 38 °C (Figure S1, Supporting Information).

Leveraging these structure–property relationships, dodecanol was identified as the optimal liquid phase. This selection strategically balances three key requirements: (i) it effectively regulates PBMA chain entanglements to achieve superelasticity (Dynamic Chain Modulation); (ii) it maintains appropriate interfacial interactions between the alkanol and polymer matrix (Interaction Optimization); (iii) it preserves electric-field control over the conductive CNT network (Electrical Responsiveness). Thus, lauryl alcohol establishes the molecular foundation for fabricating multifunctional composite organogels, exhibiting integrated superelasticity, hydrophobicity, and electric-field responsiveness.

### 2.2. Characterization of Physical Properties of the PL Oleogel

**Figure 2:**
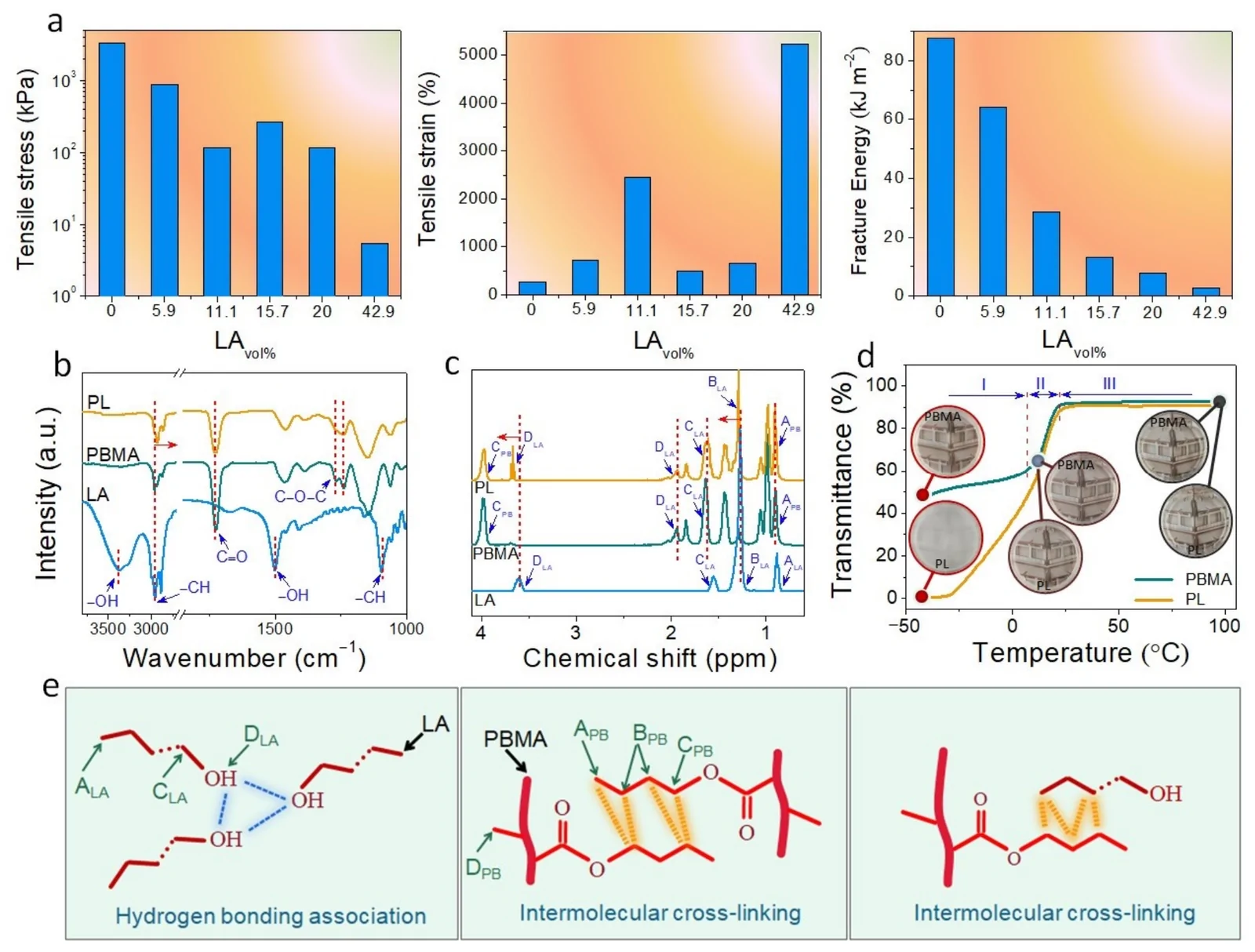
Characterization of physical properties. (**a**) Tensile performance characterization. (**b**) FT–IR spectrum of the LA, PBMA and PL oleogel. (**c**) ^1^H CP/MAS NMR. (**d**) Visible light transmittance curves of PBMA and PL samples at different temperatures. Note: Region I, II and III represent the glassy state, transition zone, and high elastic state, respectively. (**e**) Intermolecular interaction in PBMA/LA oil gel.

Figure 2a illustrates the tensile behavior of the PL oleogel in relation to its composition. Adjusting the volume fraction of lauryl alcohol (LA vol%) significantly enhanced the ductility of the PBMA polymer. At 11.1 vol% LA, the composite achieved an elongation at break of 2460%, representing an order-of-magnitude increase over pure PBMA polymer (260%). This enhancement stems from the disentangling effect of LA’s linear alcohol chains on the inter-chain entanglement network within the PBMA molecular structure. Notably, the material’s fracture tensile strength is inversely correlated with increasing liquid phase content. The fracture tensile strength of the PL oleogel containing 11.1 vol% LA decreased to 120 kPa, merely 3.5% of the pure PBMA polymer’s strength. Note that excessive LA significantly dilutes the main chain of PBMA polymer, leading to the failure of the composite’s resilience (42.9 vol% LA).

To elucidate the interaction mechanism between LA and PBMA, molecular-scale characterization was performed using Fourier transform infrared spectroscopy (FT–IR) and ^1^H CP/MASNMR. FT–IR analysis revealed hydrogen bonding association at the terminal hydroxyl group of pure LA molecules, evidenced by characteristic peaks at 3376 cm^−1^ (stretching vibration) and 1498 cm^−1^ (bending vibration) (Figure 2b and Figure S2, Supporting Information). These peaks disappeared in the PL gel system with a 11.1 vol% LA. A similar phenomenon was observed in the PBMA/TD composite system (Figure S3, Supporting Information). Further analysis showed a shift in the –CH stretching vibration peak from 2945 cm^−1^ in pure components to 2915 cm^−1^ in the gel phase, accompanied by the disappearance of the –CH bending vibration peak at 1098 cm^−1^. These changes confirm molecular interaction between LA and PBMA carbon chains. Critically, the ester groups of PBMA (–O = C and –C–O–C–) showed no significant displacement in either the PBMA/LA or PBMA/TD systems, indicating these groups do not participate in interactions with the terminal hydroxyl groups of the linear alcohols. ^1^H NMR results (Figure 2c) revealed a downfield shift in the signal peak corresponding to LA’s terminal hydroxyl group (D_LA_) from 3.59 ppm in the pure sample to 3.66 ppm in the PL oleogel. Concurrent downfield shifts were also observed for LA’s carbon chain signals (B_LA_, C_LA_). Collectively, these spectral shifts confirm the elimination of hydrogen bonding association at LA’s terminal hydroxyl group and the formation of LA–PBMA molecular chain entanglements within the PL gel, as depicted in the mechanistic model shown in Figure 2e.

We systematically characterized the evolution of PBMA–LA interactions across temperatures through visible light transmittance measurements and cooling curve analysis. As a typical thermoplastic polymer, PBMA exhibits excellent optical transparency at temperatures ≥ 22.5 °C, maintaining a stable visible light transmittance of 91 ± 0.5% (Figure 2d). When ambient temperatures decreased to the range of 7.3–22.5 °C, the transmittance of PBMA films displayed significant temperature dependence, correlating positively with rising temperatures. Notably, within the glassy state (Region I, temperatures < 7.3 °C), the material’s transmittance stabilized at a constant level of 53 ± 6%. The optical properties of PL oleogels in the high-elasticity state (Region III) and transition zone (Region II) closely aligned with those of pure PBMA. In stark contrast, upon entering the glassy state, the visible light transmittance of PL oleogels decreased sharply as temperatures dropped. Transmittance approached zero at approximately −30 °C, which was visually confirmed by embedded digital photographs. Cooling curve analysis (Figure S4, Supporting Information) further revealed phase transition behavior. Pure lauric acid exhibited a distinct isothermal plateau at 22.5 °C, corresponding to its solid–liquid phase transition. For the PL oleogel with a 11.1 vol% LA, this phase transition plateau shifted downward to 3.2 °C. At temperatures above 3.2 °C, intermolecular PBMA–LA interactions significantly surpassed LA–LA self-association, endowing the PL composite with hyperelastic mechanical characteristics (Figure S5, Supporting Information). Conversely, dominant LA–LA interactions induced crystallization at below 3.2 °C, causing the composite to transition to rigid mechanical behavior (Figure S6, Supporting Information). These findings provide critical experimental insights into the temperature-responsive mechanisms of polymer–solvent composite systems.

### 2.3. Characterization of Physical Properties of the PBMA/LA/CNTs Oleogel

**Figure 3:**
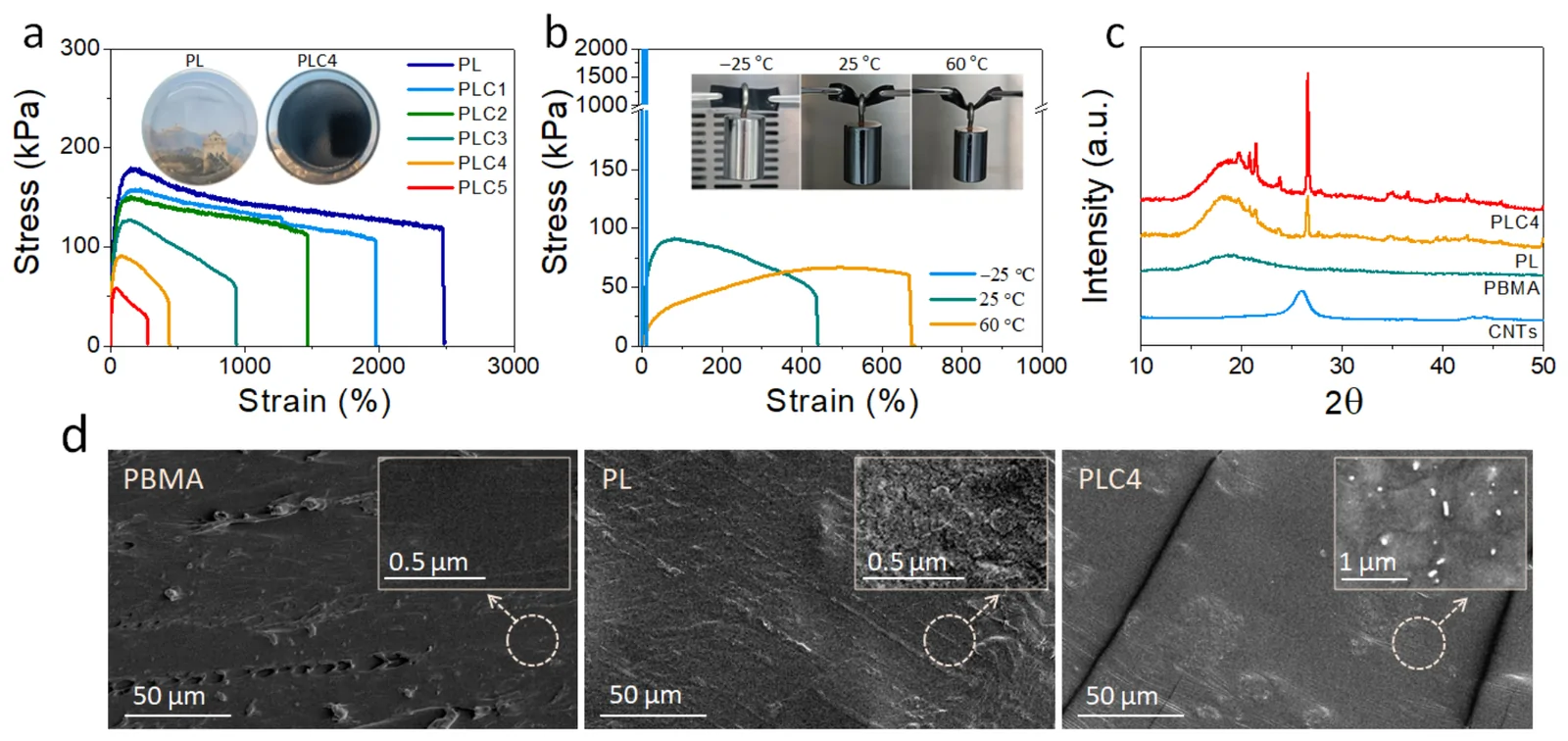
Characterization of physical properties. (**a**) Stress–strain curves of the PL and PLC*x* (*x* = 1–5). (**b**) Stress–strain curves of the PLC4 composite at different temperatures. (**c**) XRD spectrum of the CNTs, PBMA, PL and PLC4 samples. Note: the test temperature is 25 °C, and pure LA straight chain alcohol is in a molten state. (**d**) FE–SEM images of the PBMA, PL and PLC4 samples. Note: the distribution of carbon nanotubes in fresh PLC4 sample is uneven.

To enhance the electrical conductivity of PBMA/LA organogels, commercially available multi-walled carbon nanotubes were incorporated as functional fillers. The stress–strain behavior of the PLC*x* (*x* = 1–5) composites is presented in Figure 3a. As the CNTs loading increased, both the fracture stress and fracture strain of the PLC*x* organogels exhibited a concurrent decreasing trend. For instance, the PLC4 sample with a CNTs mass ratio of 205:23:1, demonstrated a fracture stress of 41.3 kPa and a fracture strain of 437%. These values accounted for 34.4% and 17.8% of the corresponding values for the pure PL organogel, indicating a significant and dramatic compromise in mechanical performance.

To elucidate the mechanism behind the degradation in mechanical properties of PL organogels induced by multi-walled carbon nanotubes, PBMA, PL, and PLC4 samples were further characterized using FT–IR, X-ray diffraction (XRD), and field emission scanning electron microscope (FE–SEM). As shown in Figure S7, the characteristic FT–IR bands of PL and the PLC4 composite overlap significantly, particularly the absorption peaks corresponding to –CH, –C = O, and –C–O–C– functional groups, showing no noticeable shifts. This indicates that CNTs are primarily dispersed within the PL organogel matrix through physical mixing. Pure PBMA polymer exhibited an amorphous structure with no distinct crystalline diffraction peaks detected (Figure 3c). However, upon the introduction of LA, well-defined crystalline diffraction peaks emerged at 21.5°, 26.7°, 36.5°, and 42.4°. Given that LA’s crystallization temperature is below 24 °C (Figure S4, Supporting Information), the crystalline peaks observed in the PL sample are attributable to the PBMA polymer. FE–SEM imaging (Figure 3d) revealed a dense nanostructure of particles (diameter < 200 nm) on the fresh fracture surface of the PL composite, distinctly contrasting with the smooth fracture morphology of pure PBMA. This clearly confirms LA’s role in inducing the crystallization of PBMA. The incorporation of CNTs further enhanced the crystallinity of PBMA, evidenced by the increased intensity of the corresponding crystalline diffraction peaks observed in XRD (Figure 3c). Nevertheless, structural defects are likely to exist at the interfaces between the crystalline domains and the glassy domains, generating residual internal stresses. This microstructural alteration, induced by the enhanced crystallinity, is identified as the intrinsic mechanism responsible for the decline in the macroscopic mechanical performance of the hyperelastic matrix. Furthermore, FE–SEM images revealed non-uniform dispersion of CNTs within the PBMA/LA organogel (Figure 3d), a factor that may also contribute detrimentally to the mechanical properties.

The effect of temperature on the mechanical properties of the PLC4 composite is shown in Figure 3b. After thermal treatment at 60 °C for 24 h, the fracture strain of the sample significantly increased to 671%, representing a 55.0% improvement compared to room temperature conditions. Conversely, the corresponding fracture stress decreased by approximately 22.0%. In contrast, when the temperature was reduced to −25 °C, the combined effects of the glass transition of the PBMA segments and the crystallization of linear LAs transformed the PLC4 composite from an elastomer into a rigid material. Embedded digital photographs visibly confirm the pronounced regulatory effect of temperature on the material’s macroscopic mechanical state. These results demonstrate that the performance of the CNT-reinforced PL organogel composites is strongly dependent on their composition, microstructure, and external temperature.

**Figure 4:**
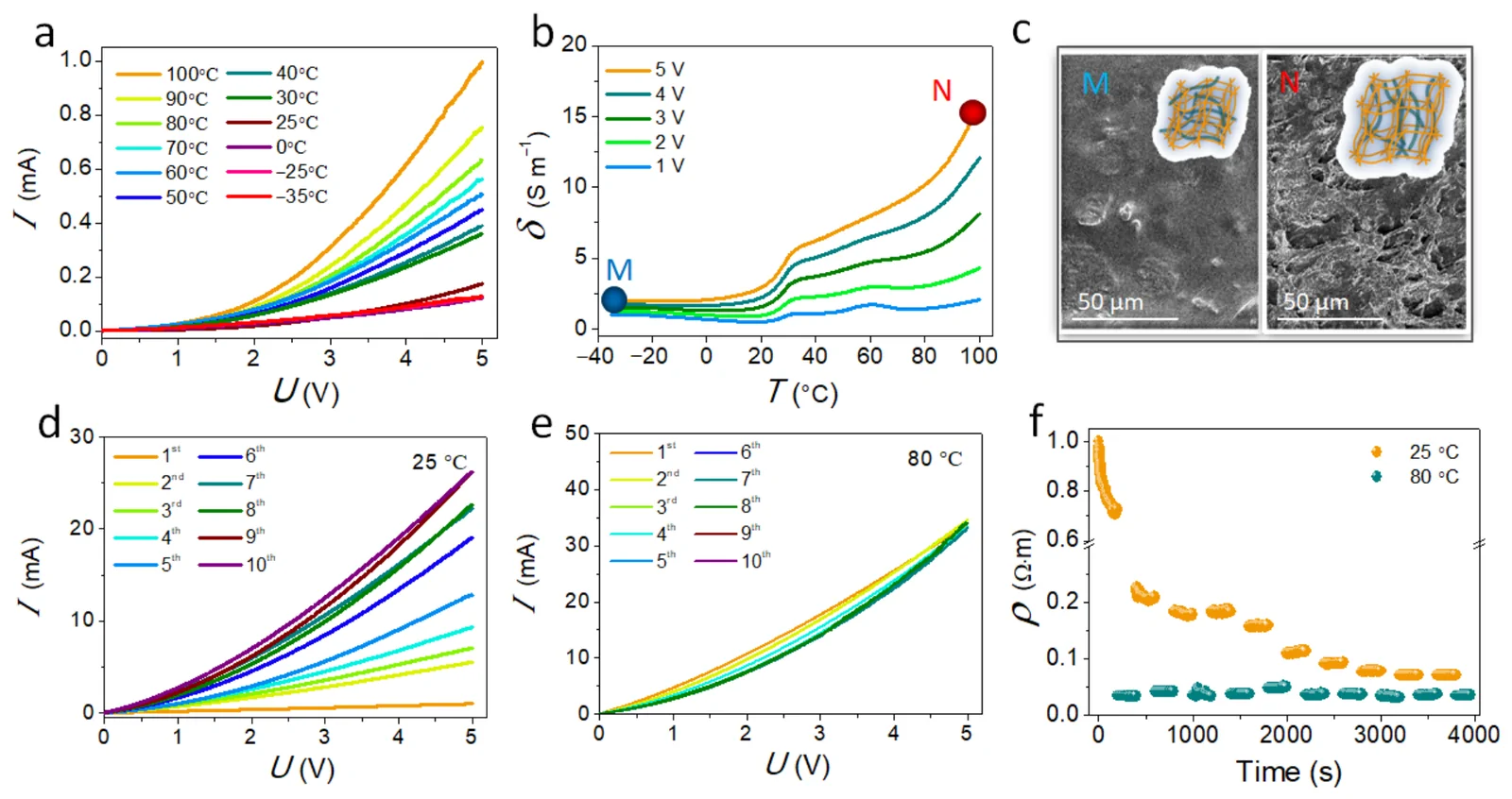
Electrical performance characterization of the as-prepared PLC4 oleogel. (**a**) LSV curves of the PLC4 at different temperatures. (**b**) *δ*–*T* curves of the PLC4 composite. Note: Blue M, the sample was frozen at −35 °C; Red N, the sample was subjected to 100 °C high-temperature treatment. (**c**) FE–SEM images of the PLC4. Note: the distribution of CNTs remains uneven at *M* point. However, its distribution at *N* point is already very uniform. (**d**) LSV curves of the PLC4 at 25 °C in thermal cycling experiment. (**e**) LSV curves of the PLC4 at 80 °C in thermal cycling experiment. (**f**) Resistivity variation curve of the PLC4.

This study systematically characterized the electrical properties of the PLC4 organogel. Figure 4a presents the linear sweep voltammetry curves of the PLC4 composite gel measured under varying temperature conditions. Distinct from electronic components exhibiting constant resistance, the composite gel demonstrated an approximately quadratic functional dependence of the signal current (*I*) on the applied voltage (*U*) at constant temperature (Figure S7, Supporting Information), deviating from typical ohmic behavior. Concurrently, the signal current exhibited significant temperature dependence, increasing with rising ambient temperature under a constant operating voltage. This phenomenon indicates that the electrical response of the carbon nanotube conductive network within the PLC organogel is regulated by the dual influence of external electric field strength and environmental temperature.

Figure 4b illustrates the temperature dependence of the normalized conductivity (*δ*) for the PLC4 organogel, using its conductivity at −35 °C and 1 V as the baseline. When the ambient temperature falls below 20 °C, the PBMA elastic three-dimensional network within the composite gel undergoes a glass transition. This state significantly diminishes the ability of the electric field to modulate the conductive network formed by multi-walled carbon nanotubes. As the temperature rises above 25 °C, the PBMA three-dimensional framework recovers its elastomeric state, and the composite gel’s conductivity exhibits a stepwise increase. For instance, under a constant working voltage of 5 V, the *δ* values for the PLC4 composite at −35, 0, 25, 50 and 100 °C are 2.0, 1.9, 2.8, 7.1, and 15.8 S·m^−1^, respectively. Within the elastomeric temperature range, *δ* also displays a pronounced voltage dependence. At a constant temperature of 100 °C, *δ* reaches 2.1, 4.3, 8.1, 12.1, and 15.8 S·m^−1^ under working voltages of 1, 2, 3, 4 and 5 V, respectively. This clearly demonstrates that the conductive properties of the PLC composite gel in the elastomeric state are synergistically regulated by both temperature and electric fields. When the temperature exceeds 60 °C, the intra- and intermolecular crosslinking capability of PBMA molecules is markedly impaired, triggering a phase transition from an elastic state to a viscous state. In this viscous regime, CNTs exhibit enhanced mobility and tend to rearrange under external forces, which further optimizes the constructed conductive network. This structural evolution is macroscopically manifested as a sharp elevation in electrical conductivity. This promotes their reorganization into a more refined and continuous three-dimensional conductive pathway. The microstructural morphology images provided in Figures S8 and S9, Supporting Information offer visual confirmation of the dynamic redistribution of CNTs within the composite gel matrix under the combined influence of temperature and the electric field. These images serve as direct structural evidence supporting the aforementioned synergistic regulation mechanism.

To thoroughly investigate the electrical stability of PLC4 composite gels, this study systematically examined their electrochemical behavior under thermal cycling conditions ranging from 25 °C to 80 °C. Electrical response data, acquired through linear sweep voltammetry and constant-current voltage tests, were normalized and comprehensively presented in Figure 4d–f. At an ambient temperature of 25 °C, the response current intensity of the PLC4 composite gel exhibited a progressive enhancement with increasing thermal cycles. Under a constant 5 V bias, the response currents after the fifth and tenth thermal cycles reached 12.8 and 26.1 mA, respectively. These observations indicate that the synergistic interaction between thermal and electric fields effectively reconstructs the topological structure of the CNT conductive network. This optimization effect demonstrates sustained gains even at room temperature. Remarkably, the *I*–*U* characteristic curves remained highly consistent at 80 °C after multiple thermal cycles (Figure 4e), confirming a significant structural memory effect within the CNT conductive network under high-temperature conditions. As further illustrated in Figure 4f, resistivity evolution mapping corroborates the controllable modulation mechanism of the CNT network by thermal and electric fields. The resistivity values gradually converged and stabilized as cycling progressed, robustly demonstrating the exceptional electrical stability and structural reliability of the PLC composite gel under thermal cycling. This characteristic highlights its promising potential for applications in environments with fluctuating temperatures.

**Figure 5:**
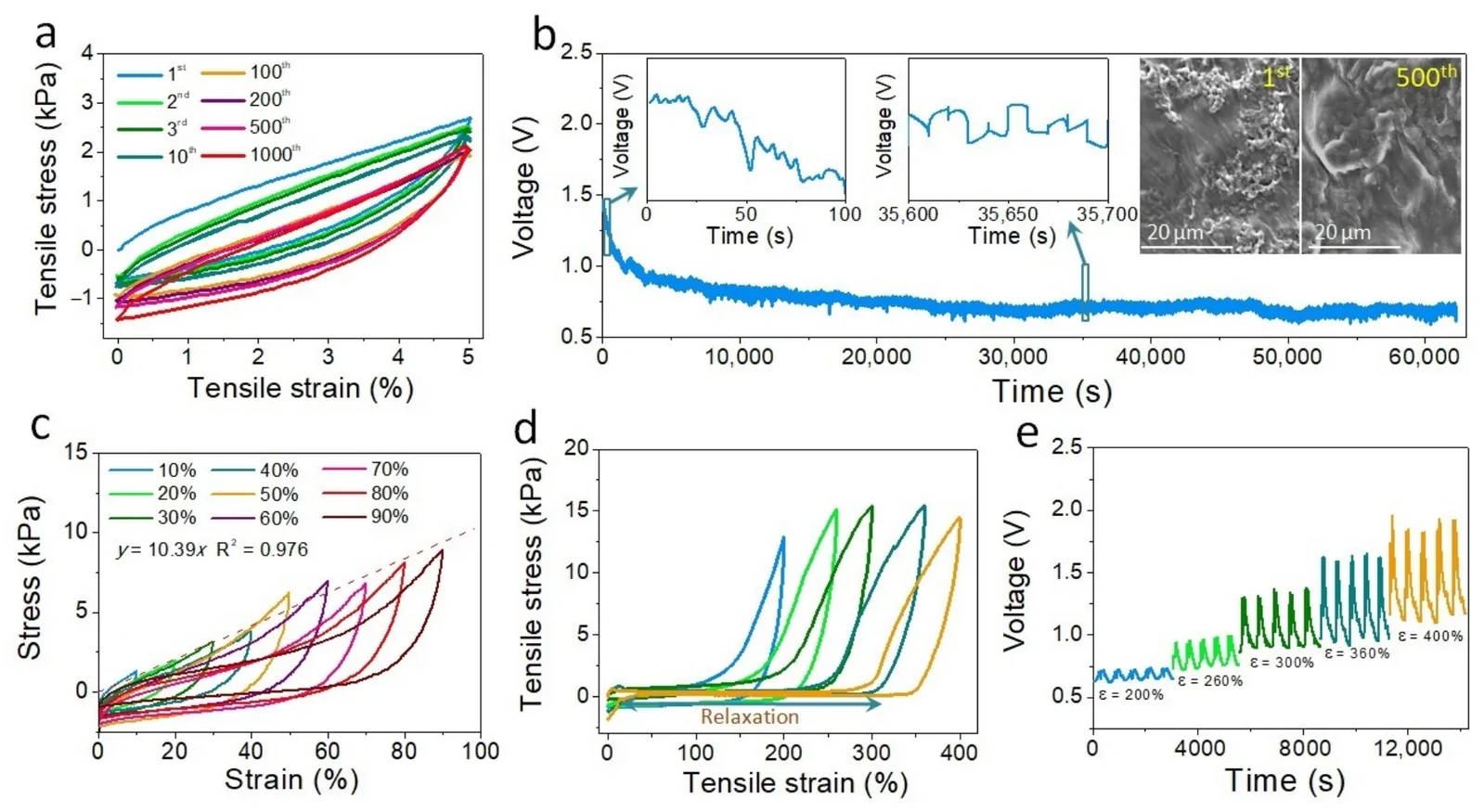
Electrical behavior of PLC4 composite gel in tensile state. (**a**) The stress–strain curves of PLC4 composite under 5% micro tensile strain. (**b**) Dynamic electrical behavior of PLC samples under 1000 stretching cycles. Note: Embedded image shows the microstructure of the composite before/after stretching. (**c**,**d**) Mechanical properties of the PLC4 under different tensile strains. Note: The dashed line in Figure c is the linear regression fitting line representing the relationship between the maximum stress and the strain. (**e**) Electrical signal response during stretching cycle process.

To further explore the sensing characteristics of the PLC composite gel, this study conducted in situ testing to analyze its stress–strain behavior and voltage response signals during cyclic stretching. Figure 5a displays the cyclic loading curves of the composite gel within the micro-strain range (tensile strain ≤ 5%). Even after 1000 cycles, the morphology of the stress–strain curves remained largely unchanged, demonstrating the material’s exceptional mechanical sensitivity and cyclic stability. Notably, the fresh PLC4 composite in its initial state failed to generate regular voltage signals during stretching (Figure 5b). This phenomenon can be attributed to the non-uniform dispersion of CNTs within the composite (Figure 3d), which prevented the effective formation of a 3D conductive network. After approximately 500 stretching cycles, however, the nanotubes underwent stress-induced reorientation and rearrangement (embedding diagram in Figure 3). Accompanying this structural evolution, the signal voltage significantly decreased from 1.46 V in the initial cycle to 0.68 V and exhibited a periodic response highly synchronized with the tensile strain. This voltage evolution trend clearly reveals the modulation mechanism of directional tensile stress on the CNT conductive network within the composite gel.

To thoroughly investigate the impact of strain amplitude on material behavior, we systematically tested the mechanical and electrical responses of the material under varying tensile strains. When the tensile strain was confined within 100%, a strong linear correlation emerged between the maximum stress and strain (Figure 5c). Electrical characterization revealed that under low-strain conditions (e.g., *ε* = 10% and 30%), the voltage signal exhibited no periodic characteristics (Figure S10, Supporting Information). However, as the strain amplitude increased beyond 50%, periodic oscillations synchronized with the stretching cycles became evident in the signal voltage. This phenomenon was consistently verified in Figure 5e. Consequently, the established structure–property relationship proves conclusively that directional tensile stress serves as a crucial external driving force. Beyond conventional regulatory factors like temperature and electric fields, it fundamentally governs the formation and reconstruction of the multi-walled carbon nanotube conductive network within PLC composites.

**Figure 6:**
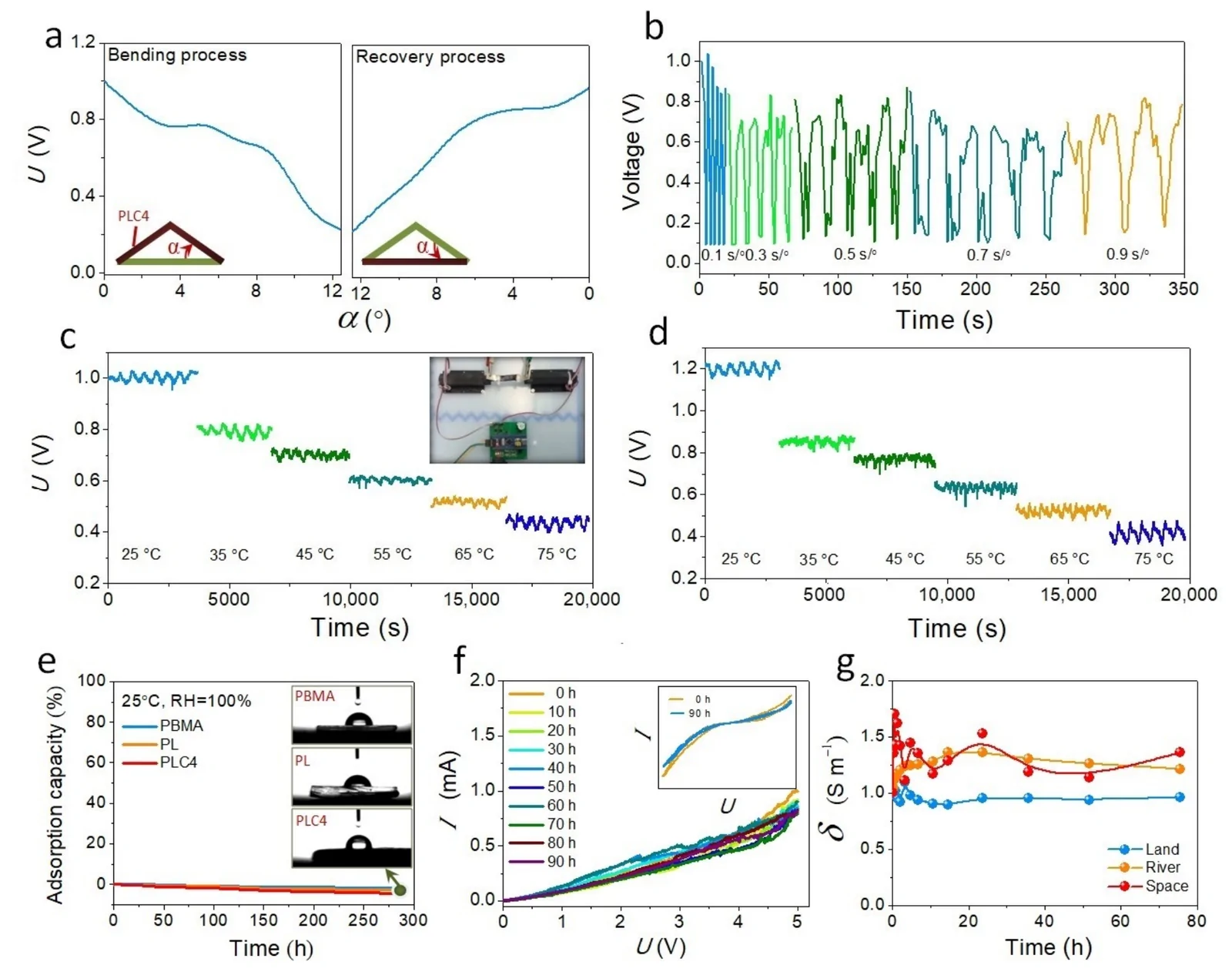
Electrical behavior of PLC4 composite gel. (**a**) Signal voltage of PLC4 composite gel at different bending angles. Note: The arrow indicates the rotational direction of the PLC4 composite sample during the bending resistance test. (**b**) Electrical signal response curve of the PLC4 composite under different bending frequencies. (**c**) Relationship curve between temperature and signal voltage of the PLC4 composite at standard atmospheric pressure. Note: The embedded image shows a self-made tensile testing device. (**d**) *U*–*T* of the PLC4 sample under vacuum negative pressure. (**e**) Adsorption curves of the PBMA, PL and PLC4 samples at 25 °C during the relative humidity of 100%. Note: The embedding diagram is the infiltration angle test diagram. (**f**) LSV and CV curves of the PLC4 sample. (**g**) *δ*–*t* curves of the PLC4 composite under different humidity and pressure environments.

Further analysis reveals the electrical response characteristics of PLC4 composite gel under bending stress. Notably, test specimens were pre-treated via synergistic electric and thermal field modulation to optimize the structural integrity of their internal carbon nanotube conductive networks. Under constant current conditions, the output signal voltage of the composite gel increases incrementally with bending angle (*α*). During a single bending-recovery cycle, the signal voltage variation curve exhibits significant axisymmetric behavior (Figure 6a). Figure 6b illustrates the dynamic voltage response across varying rotational frequencies. The high repeatability of signals at the same frequency, coupled with minimal amplitude fluctuations between different frequencies, demonstrates the dual ability of materials to exhibit sensitivity and stability under dynamic bending deformation. Figure 6c and d characterize the relationship between electrical signals and tensile cycles under standard atmospheric pressure and vacuum conditions, respectively. At constant temperature, signal voltage displays periodic oscillations synchronized with tensile cycles. This behavior indicates negligibl influence of environmental pressure on conductive network performance, suggesting potential applicability in aerospace extreme environments.

From a compositional perspective, poly(butyl methacrylate), dodecanol, and multi-walled carbon nanotubes all exhibit hydrophobic properties, endowing PLC composite gel with intrinsic moisture resistance. Experimental validation was obtained through water vapor adsorption isotherms and contact angle measurements (Figure 6e). To further investigate water tolerance, PLC4 gel was immersed in deionized water for electrical characterization. Linear sweep voltammetry and cyclic voltammetry curves showed nearly identical scanning trajectories (Figure 6f). Quantitative conductivity comparisons across environments (Figure 6g) confirm stable conductivity values before and after immersion, proving minimal impact of water infiltration on conductive networks. This establishes a foundation for functional reliability in humid environments (e.g., hydrologic monitoring, aquatic electrophysiology) and liquid media.

## 3. Conclusions

In this study, we present a simple design strategy for fabricating a novel conductive organogel featuring fully physical crosslinks. The long alkyl chains of LA form physical crosslinks with the side chains of PBMA. This unique structure effectively replaces the inherent chain entanglements within PBMA itself. This innovative mechanism significantly facilitates the rapid rotation and slippage of PBMA chains during stretching, endowing the organogel with remarkable hyperelasticity, achieving elongations exceeding 2460%. Concurrently, the incorporated carbon nanotubes (CNTs) undergo displacement or rotation under the synergistic effects of temperature and electric fields, leading to the formation of a more complete conductive network. This distinctive structural design results in a composite organogel exhibiting a conductivity at 100 °C that is 15 times higher than its conductivity at room temperature. Crucially, the PLC composite organogel demonstrates highly reliable flexibility and stable electrical performance even under demanding conditions, including prolonged immersion in deionized water, exposure to vacuum, or high-temperature environments up to 120 °C. This novel design successfully overcomes inherent limitations typically associated with traditional polymer hydrogels, such as mechanical properties heavily dependent on ambient humidity, poor tolerance to high temperatures (usually below 80 °C), and vulnerability to dehydration. It thereby offers a new conceptual framework for the design of highly stretchable gel-based materials.

## 4. Materials and Methods

### 4.1. Material

All chemical reagents utilized in this study were procured at analytical reagent grade and employed without additional purification. The experimental materials included butyl methacrylate (≥98.0%), ethylene glycol dimethacrylate (≥99.0%), azobisisobutyronitrile (AIBN, ≥98.0%), lauryl alcohol (≥99.98%), and multi-walled carbon nanotubes (≥95%). These materials were sourced from Aladdin Biochemical Technology Co., Ltd. (Shanghai, China), a NASDAQ-listed enterprise (SSE: 688179) specializing in high-purity chemical reagents with ISO-certified manufacturing facilities.

### 4.2. Preparation of the PLC Composites

Preparation PL Organogel: A homogeneous solution was prepared by dissolving *x* ml of lauryl alcohol and 16.0 mL butyl methacrylate under constant magnetic stirring at 50 °C (Figure 1). Subsequently, 0.032 g of AIBN was added as an initiator and 0.104 mL of EGDMA was used as a crosslinking agent to obtain a transparent mixed solution. The uniformly stirred suspension was poured into a culture dish and aged for 24 h in a 75 °C blast drying oven to obtain stable PL*_x_* organogel materials (Table 1).

Preparation PLCy Organogel: A homogeneous solution was prepared by dissolving 2.0 mL of lauryl alcohol and 16.0 mL butyl methacrylate under constant magnetic stirring at 50 °C. Subsequently, 0.032 g of AIBN was added as an initiator and 0.104 mL of EGDMA was used as a crosslinking agent to obtain a transparent mixed solution. Controlled quantities of CNTs (*m* = 0.5, 1.0, 1.5, 2.0 and 2.5 g) were introduced into the BMA/LA mixed solution. The uniformly stirred suspension was poured into a culture dish and aged for 24 h in a 75 °C blast drying oven to obtain stable PLC*_y_* composite material (Table 1).

### 4.3. Material Characterization

Microstructure and Chemical Analysis: The microstructure of the frozen fracture sample surface was observed using a field emission scanning electron microscope (Hitachi S4800, Tokyo Hitachi, Ltd., Tokyo, Japan). To avoid charge effects, the sample was treated with gold spray before observation, with an acceleration voltage of 5 kV. Fourier transform infrared spectroscopy (Nicolet 5700, Waltham Thermo Fisher Scientific Inc., Waltham, MA, USA) was used to analyze chemical functional groups and intermolecular interactions. The spectral recording range is 4000–500 cm^−1^, with a resolution of 4 cm^−1^, using KBr compression method. Grind the freeze-dried gel sample with spectral pure KBr powder, press it into a transparent sheet, and then measure it. The X-ray diffraction pattern was collected on a Bruker D8 Advance X-ray diffractometer (Bruker D8 Advance, Karlsruhe Bruker AXS GmbH, Karlsruhe, Germany) using a Cu K α radiation source (λ = 0.154056 nm) with a working voltage of 40 kV and a current of 40 mA. The scanning range was 2*θ* from 10° to 50°, with a step size of 0.02° and a scanning rate of 4°min^−1^, used for analyzing crystal structures. The solid-state 1H CP/MAS NMR spectrum was recorded on a JEOL JNM–ECZ-600R spectrometer with a resonance frequency of 600 MHz, used to study the intermolecular interactions between PBMA and LA. The sample is loaded into a 4 mm zirconia rotor with a magic angle rotation rate of 12 kHz.

Mechanical Performance Testing: The tensile test was conducted at 25 °C using an electronic universal testing machine (WDW–100C, Shanghai Songdun Instrument Manufacturing Co., Ltd., Shanghai, China). The sample is cut into rectangular strips with dimensions of 2 mm (thickness) × 4 mm (width) × 20 mm (length), and the gauge length is set to 10 mm. The test is conducted at a constant beam speed of 100 mm min^−1^, and the force value is recorded using a 100 N force sensor. The strain is calculated from the displacement of the beam. To study the effects of temperature, PLC4 samples were tested at different temperatures, and the samples were kept at the set temperature for 30 min before testing. At least 5 samples should be tested for each condition to ensure reproducibility, and the results should be expressed as mean ± standard deviation.

Optical and Thermal Analysis: Measure the visible light transmittance of samples (PBMA, PL, and PLC4) in the wavelength range of 380–760 nm using an LS116 transmittance meter (Shenzhen Linshang Technology Co., Ltd., Shenzhen, China). The testing temperature range is −40 °C to 60 °C, and the temperature is controlled by a programmable temperature control box. Record the cooling curve to analyze the phase transition behavior of LA and PL/PLC4 composites. Heat the sample to 60 °C to ensure complete melting, then cool it to −20 °C at a constant rate of 1 °C min^−1^. The temperature is recorded every 2 s by a multi-channel temperature recorder (AT4516, Changzhou Anbai Precision Instrument Co., Ltd., Changzhou, China).

Electrical Performance Testing: The electrical properties of PLC composites were characterized using a CHI660E electrochemical workstation (Shanghai Chenhua Instrument Co., Ltd., Shanghai, China). Linear sweep voltammetry is performed within the voltage range of −5 V to 5 V at a scan rate of 100 mV s^−1^. Cyclic voltammetry is performed within the same voltage range at a scan rate of 50 mV s^−1^. The chronopotentiometry method is used to record the voltage time response at a constant current. All electrochemical measurements were conducted in a three-electrode system, with PLC composite material (20 mm × 10 mm × 2 mm) as the working electrode, platinum plate as the counter electrode, and Ag/AgCl electrode as the reference electrode.

## Figures and Tables

**Table 1:** Composition of PL*_x_* and PLC*_y_* samples.

Sample	*V*_BMA_ (mL)	*V*_LA_ (mL)	*m*_CNTs_ (g)	Mass Fraction of CNTs (wt%)
PBMA	16.0	0	0.5	3.0
PL1	16.0	1.0	0.5	3.0
PL2	16.0	2.0	0.5	3.0
PL3	16.0	3.0	0.5	3.0
PL4	16.0	4.0	0.5	3.0
PL5	16.0	12.0	0.5	3.0
PLC1	16.0	2.0	0.5	3.0
PLC2	16.0	2.0	1.0	5.9
PLC3	16.0	2.0	1.5	8.6
PLC4	16.0	2.0	2.0	11.1
PLC5	16.0	2.0	2.5	13.5

## Data Availability

The data presented in this study are available within the article and its Additional Files.

## Supplementary Materials

The following supporting information can be downloaded at https://www.mdpi.com/article/10.3390/gels12080707/s1, Figure S1: Digital photos of PBMA/TD composite gel at different temperatures. Figure S2: FT–IR spectrum of the PBMA, PL and PLC4 samples. Figure S3: FT–IR spectrum of the TD, PBMA and PBMA/TDx (x = 4, 8 and 12) oleogels with a VTD/VPBMA vol% of x:5. Figure S4: Cooling curves of the LA, PL and PLC4. Figure S5: Digital photo of the PL sample. Figure S6: Digital photos of the PL and PLC4 composites at different temperatures. Figure S7: Electrical properties of the PLC composites. (a) LSV curve testing and fitting of PLC4 sample. (b) Conductivity of composites with different carbon nanotube contents. Figure S8: OM images of the LA/CNTs and PLC4 composite. (a) Microscopic morphology of the LA/CNTs sample. (b) Microscopic morphology of the PLC4. (c) Microscopic morphology under electric field induction. Note that all samples were characterized at 80 °C. Figure S9: FE-SEM images of the PLC4 at N point. Figure S10: Electrical signal response of PLC4 sample under different tensile strains.

## Abbreviations

The following abbreviations are used in this manuscript:

LA	Lauryl alcohol
CNTs	Multi-walled carbon nanotubes
PBMA	Poly(butyl methacrylate)
AAM	Acrylamide
ROH	Aliphatic alcohols
BMA	Butyl methacrylate
EGDMA	Ethylene glycol dimethacrylate
AIBN	Azobisisobutyronitrile
FE–SEM	Field emission scanning electron microscope
XRD	X-ray diffraction
FT–IR	Fourier transform infrared spectroscopy
