# Supplementary material for: Physically Crosslinked Conductive Organic Gel with Excellent Elasticity and Environmental Stability
Source: Gels. 2026 Aug 8;12(8):707. doi: 10.3390/gels12080707 (PMC13511898; doi:10.3390/gels12080707)
Supplement: Supplementary file 1 [file gels-12-00707-s001.zip › gels-4410588-supplementary.pdf]

Supporting Information

Physically Crosslinked Conductive Organic Gel with Excellent Elasticity and Environmental Stability

Haiquan Zhang *,†, Zhinan Zhao †, Shishen Lan †, Qiadong Yao, Minglei Lv and Ning Wang *

State Key Laboratory of Marine Resource Utilization in South China Sea, School of Marine Sciences, Hainan University, Haikou 570228, China

* Correspondence: haiquan_zhang01@hainanu.edu.cn (H.Z.); wangn02@foxmail.com (N.W.)

† These authors contributed equally to this work.

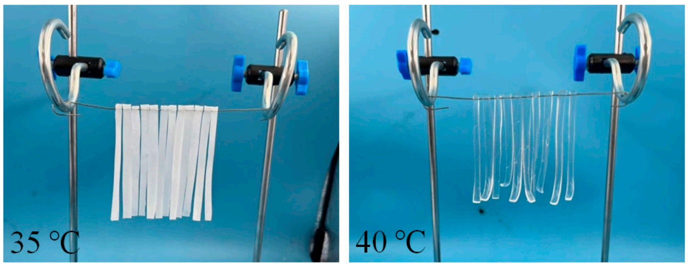

Figure S1 Digital photos of PBMA/TD composite gel at different temperatures.

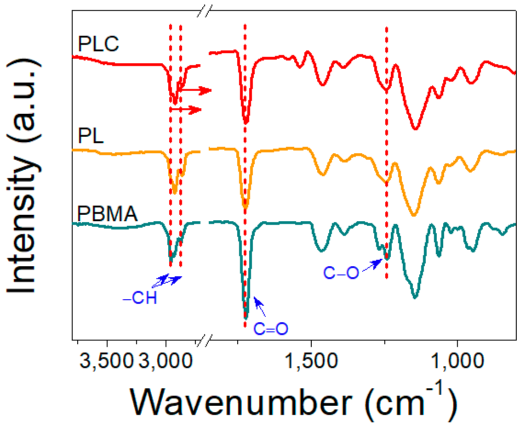

Figure S2 FT–IR spectrum of the PBMA, PL and PLC4 samples.

The introduction of carbon nanotube filler will destroy the continuous entanglement network of PBMA/LA matrix and improve the crystallinity of the system, but at the same time, it is also easy to produce interfacial structural defects due to agglomeration, resulting in synchronous attenuation of

fracture stress and fracture strain of composite oil gel. Heat treatment can weaken the intermolecular interactions of polymer chains, release internal stresses, and promote the directional rearrangement of carbon nanotubes under an electric field, effectively alleviating the deterioration of mechanical properties.

This study selected PLC4 as the core research sample and achieved the optimal balance between mechanical stretchability and conductive sensing performance based on its carbon nanotube filling amount. Specifically, PLC1-PLC3 samples with lower carbon nanotube content have weak electrical response sensitivity due to insufficient connectivity of the conductive network; however, PLC5 samples with higher filling amounts experience increased mechanical damage and poorer material formability due to the exacerbation of carbon nanotube aggregation. In contrast, PLC4 achieves the best balance in terms of mechanical flexibility, electrical response sensitivity, thermal cycling stability, and microstructure characterization typicality by constructing continuously adjustable carbon nanotube conductive pathways while also considering the considerable tensile deformation capability. This choice not only ensures the deformation ability required for the material as a flexible electronic skin, but also fully demonstrates the functional characteristics of the CNT conductive network under temperature electric field synergistic regulation, and provides a complete experimental basis for revealing the structure performance relationship of the material.

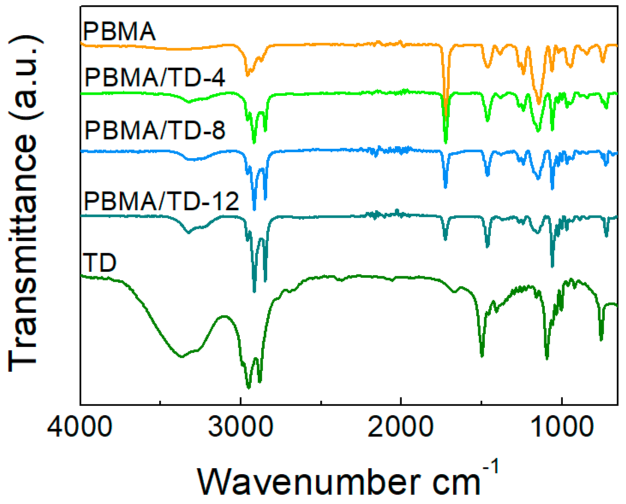

Figure S3 FT-IR spectrum of the TD, PBMA and PBMA/TD*x* (x=4, 8 and 12) oleogels with a V_{TD}/V_{PBMA} volume ratio of x:5.

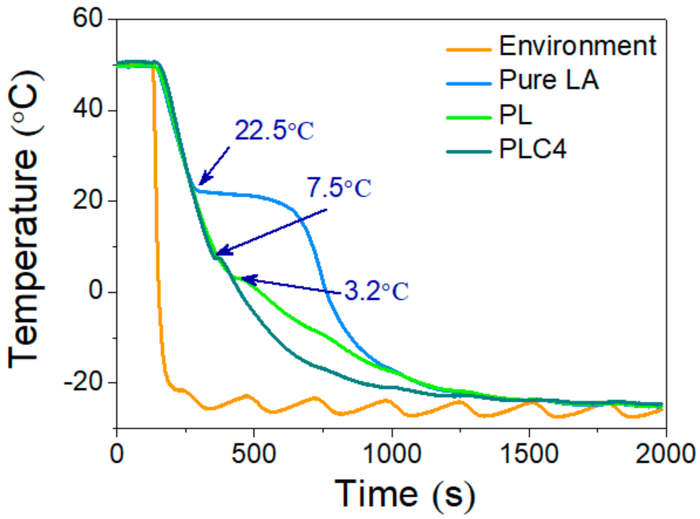

Figure S4 Cooling curves of the LA, PL and PLC4.

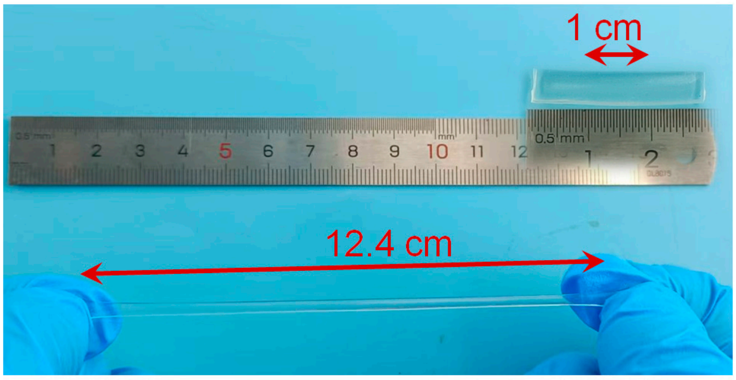

Figure S5 Digital photo of the PL sample.

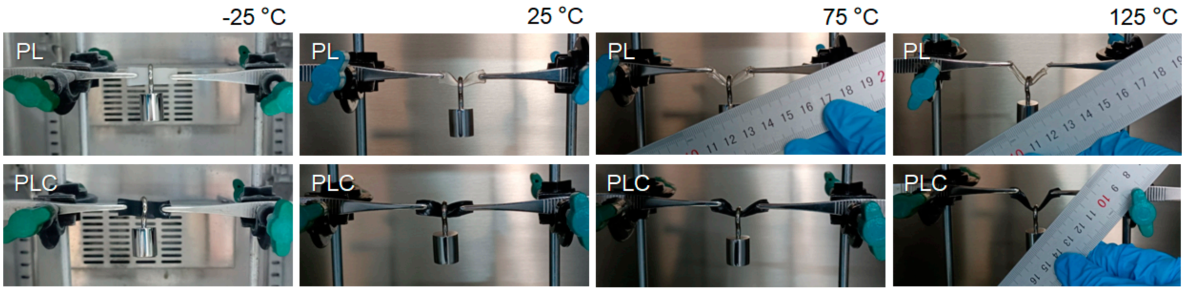

Figure S6 Digital photos of the PL and PLC4 composites at different temperatures.

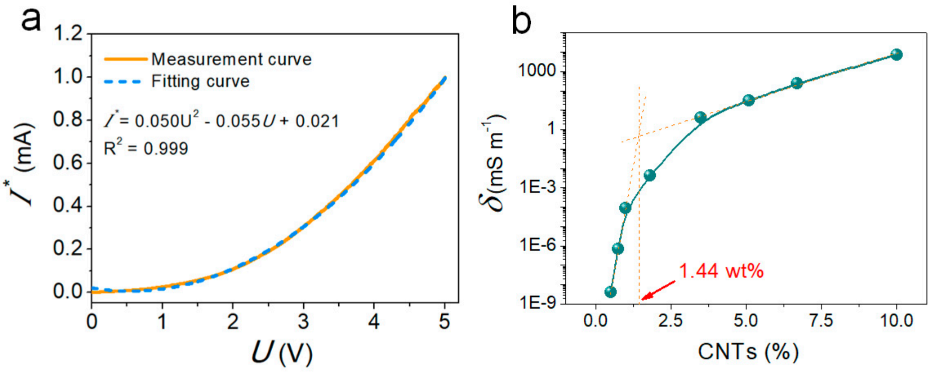

Figure S7 Electrical properties of the PLC composites. (a) LSV curve testing and fitting of PLC4 sample. (b) Conductivity of composites with different carbon nanotube contents.

The non-ohmic conductive behavior of the PLC4 composite organogel originates from the dynamic reconfiguration of its internal carbon nanotube (CNT) conductive network. As the temperature elevates above 25 °C, the intermolecular interactions between the PBMA matrix and LA molecules are weakened, which reduces the physical constraint imposed on the embedded CNTs. Under the stimulus of an external electric field, the CNTs undergo directional rotation and migration, driving the rearrangement of dispersed or poorly connected CNT segments to construct an optimized 3D conductive pathway. This electric-field-induced topological evolution of the CNT network exhibits inherent nonlinear characteristics, resulting in an approximately quadratic dependence between current (I) and voltage (U) (Figure S7, Supporting Information). Macroscopically, this manifests as a mismatch between voltage amplification and current amplification, alongside a non-monotonic increase in conductivity with rising applied voltage.

Furthermore, temperature modulates the mobility of CNTs by tuning the strength of the PBMA-LA interaction, leading to distinct responses of the CNT network to electric fields at different thermal conditions. At temperatures below 20 °C, the PBMA matrix undergoes a glass transition, which restricts the motion of polymer chains and elevates the migration resistance of CNTs, thus suppressing the nonlinearity of the I-U curve. In the high-elastic regime above 25 °C, the intermolecular interactions of both PBMA-LA and PBMA-PBMA are progressively weakened, endowing the CNTs with enhanced capability for directional migration and rearrangement under an electric field, which amplifies the nonlinear response. Experimental data confirms that the signal current increases significantly with temperature (Figure 4a). For instance, at a constant operating voltage of 5 V, the conductivity of PLC4 at 100 °C reaches 15.8 S·m^{-1}, which is 5.6 times that measured at 25 °C (2.8 S·m^{-1}). The nonlinear slope of the I-U curve also increases monotonically with the rise in temperature.

In this study, the true electrical conductivity of the as-prepared composites with different carbon nanotube (CNT) loadings was systematically measured, and the corresponding results are plotted in

Figure S7b. Based on the nonlinear variation trend of the measured conductivity against CNT content, a reliable percolation threshold of 1.44 wt% can be deduced for the obtained composite system. Further analysis on the experimental conductivity data reveals that when the CNT content does not exceed 1.8 wt%, the electrical conductivity of the composite remains below 0.0043 $mS \cdot m^{-1}$, confirming that the material exhibits typical intrinsic semiconductor characteristics.

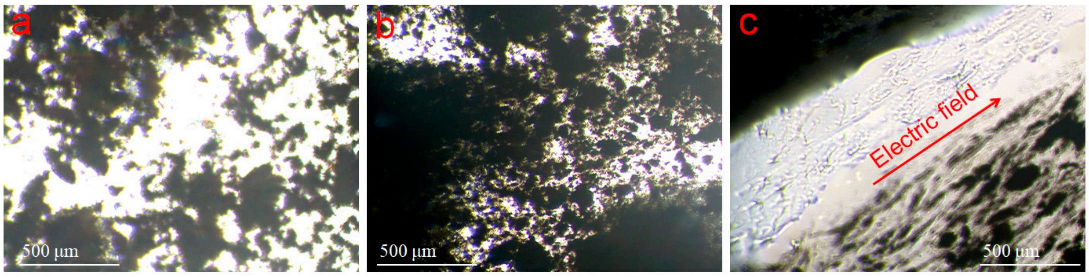

Figure S8 OM images of the LA/CNTs and PLC4 composite. (a) Microscopic morphology of the LA/CNTs sample. (b) Microscopic morphology of the PLC4. (c) Microscopic morphology under electric field induction. Note that all samples were characterized at 80 °C.

As shown in Figure S8a, CNTs are difficult to evenly distribute in LA, resulting in agglomeration phenomenon. When CNTs are added to PBMA/LA, they exhibit uniform dispersion due to the physical confinement of the PBMA polymer network. When an electric field is added at high temperatures, CNTs can undergo wonderful directional arrangement (Figure S8c).

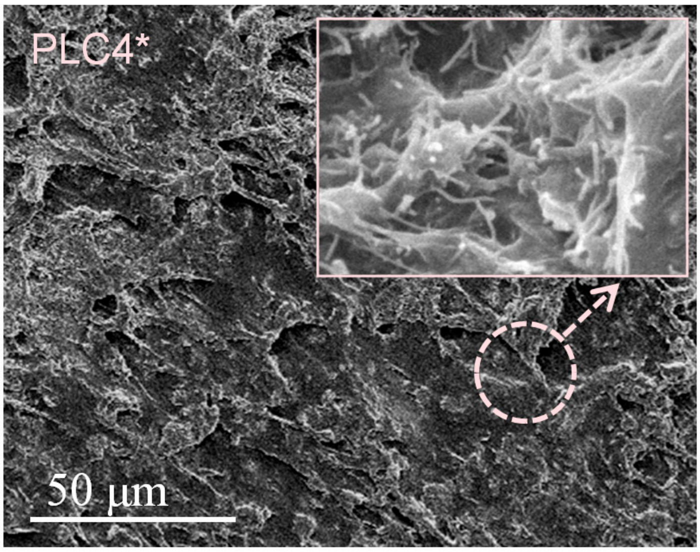

Figure S9 FE-SEM images of the PLC4 at *N* point.

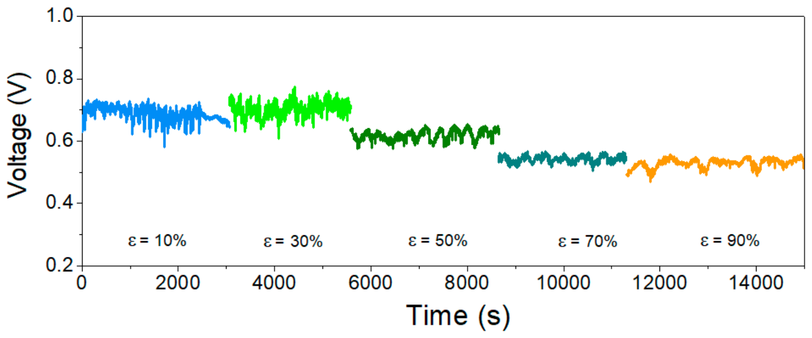

Figure S10 Electrical signal response of PLC4 sample under different tensile strains.
